# The Influence of Personality Disorder Symptoms on Treatment Outcomes in Bipolar Disorder: A Secondary Analysis of a Randomised Controlled Trial: L’influence des symptômes du trouble de la personnalité sur les résultats du traitement dans le trouble bipolaire : Une analyse secondaire d’un essai randomisé contrôlé

**DOI:** 10.1177/07067437231213558

**Published:** 2023-11-15

**Authors:** Alessandra Sarmiento, Olivia M. Dean, Bianca E. Kavanagh, Mohammadreza Mohebbi, Michael Berk, Seetal Dodd, Sue M. Cotton, Gin S. Malhi, Chee H. Ng, Alyna Turner

**Affiliations:** 1 IMPACT – The Institute for Mental and Physical Health and Clinical Translation, School of Medicine, Barwon Health, 2104 Deakin University, Geelong, VIC, Australia; 2 56369 Florey Institute for Neuroscience and Mental Health, University of Melbourne, Parkville, VIC, Australia; 3 Deakin Rural Health, 2104 Deakin University, Warrnambool, VIC, Australia; 4 Faculty of Health, Biostatistics Unit, 2104 Deakin University, Geelong, VIC, Australia; 5 Orygen, Parkville, VIC, Australia; 6 Centre for Youth Mental Health, University of Melbourne, Parkville, VIC, Australia; 7 Academic Department of Psychiatry, Kolling Institute, Northern Clinical School, Faculty of Medicine and Health, The University of Sydney, NSW, Australia; 8 CADE Clinic, Royal North Shore Hospital, Northern Sydney Local Health District, NSW, Australia; 9 Department of Psychiatry, University of Oxford, Oxford, UK; 10 Professorial Unit, The Melbourne Clinic, Department of Psychiatry, 2281 The University of Melbourne, Richmond, VIC, Australia

**Keywords:** bipolar disorder, personality disorder, treatment, clinical trial, adjunctive therapies, mania, depression, neuroscience, psychiatry, Trouble bipolaire, trouble de la personnalité, traitement, essai clinique, thérapies complémentaires, manie, dépression, neuroscience, psychiatrie

## Abstract

**Objectives:**

Many people who are diagnosed with bipolar disorder also have comorbid personality disorder. Few studies have explored how personality disorder may influence pharmacological treatment outcomes. The aim of this study was to conduct a secondary analysis of data from a clinical trial of adjunctive nutraceutical treatments for bipolar depression, to determine whether maladaptive personality traits influence treatment outcomes.

**Methods:**

Scores on the Standardised Assessment of Personality – Abbreviated Scale screener were used to classify participants as having bipolar disorder with (*n* = 119) and without (*n* = 29) above threshold personality disorder symptoms (personality disorder). Outcome measures included: The Montgomery Åsberg Depression Rating Scale, Clinical Global Impressions and Improvement Severity Scales, Patient Global Impressions–Improvement scale, Bipolar Depression Rating Scale, Range of Impaired Functioning Tool, Social and Occupational Functioning Assessment Scale and Quality of Life and Enjoyment Scale (Quality of Life Enjoyment and Satisfaction Questionnaire-Short Form). Generalised estimated equations examined the two-way interactions of personality disorder by time or treatment and investigated personality disorder as a non-specified predictor of outcomes.

**Results:**

Over time, the Patient Global Impressions–Improvement scores were significantly higher in those in the personality disorder group. No other significant differences in the two-way interactions of personality disorder by treatment group or personality disorder by time were found. Personality disorder was a significant but non-specific predictor of poorer outcomes on the Bipolar Depression Rating Scale, Range of Impaired Functioning Tool, and Quality of Life Enjoyment and Satisfaction Questionnaire-Short Form, regardless of time or treatment group.

**Conclusions:**

This study highlights the potential impact of maladaptive personality traits on treatment outcomes and suggests that the presence of comorbid personality disorder may confer additional burden and compromise treatment outcomes. This warrants further investigation as does the corroboration of these exploratory findings. This is important because understanding the impact of comorbid personality disorder on bipolar disorder may enable the development of effective psychological and pharmacotherapeutic options for personalised treatments.

## Résumé

**Objectifs:**

De nombreuses personnes qui reçoivent un diagnostic de trouble bipolaire (TB) ont également un trouble de la personnalité (TP) comorbide. Peu d’études ont exploré comment le TP peut influencer les résultats du traitement pharmacologique. La présente étude visait à mener une analyse secondaire des données d’un essai clinique de traitements nutraceutiques complémentaires pour la dépression bipolaire, afin de déterminer si les traits de la personnalité inadaptée influencent les résultats du traitement.

**Méthodes:**

Les scores à l’Échelle d'évaluation standardisée de la personnalité abrégée (SAPAS) ont servi à classifier les participants comme ayant un TB avec (n = 119) et sans (n = 29) au-dessus du seuil des symptômes de TP (« TP »). Les mesures des résultats incluaient : l’Échelle d'évaluation de la dépression de Montgomery Åsberg (MADRS), l’Échelle des impressions cliniques globales et l’échelle de gravité des améliorations (CGI-I, CGI-S), Impressions globales des patients–amélioration (PGI-I), Échelle d’évaluation de la dépression bipolaire (BDRS), Gamme de fonctionnement altéré (LIFE-RIFT), Fonctionnement social et professionnel (SOFAS) et Échelle de qualité de vie et de plaisir (Q-LES-Q-SF). Les équations estimées généralisées examinaient les interactions bidirectionnelles du TP selon le temps ou le traitement et investiguaient le TP comme prédicteur non spécifié des résultats.

**Résultats:**

Avec le temps, les scores au PGI-I scores étaient significativement plus élevés que ceux du groupe TP. D’autres différences significatives n’ont pas été trouvées dans les interactions bidirectionnelles du TP par groupe de traitement ou du TP par le temps. Le TP était un prédicteur significatif mais non spécifique de mauvais résultats aux échelles BDRS, LIFE-RIFT, Q-LES-Q-SF, peu importe le temps ou le groupe de traitement.

**Conclusions:**

La présente étude fait ressortir l’effet potentiel des traits de la personnalité inadaptée sur les résultats de traitement, et suggère que la présence d’un TP comorbide peut conférer un fardeau additionnel et compromettre les résultats du traitement. Ceci mérite plus d’investigation tout comme la corroboration de ces résultats exploratoires. C’est important parce que comprendre l’effet du TP comorbide sur le TB peut permettre de développer des options psychologiques et pharmacothérapeutiques efficaces pour des traitements personnalisés.

## Introduction

Bipolar disorder (BD) is typically a lifelong chronic disorder characterised by recurrent fluctuations in energy and mood states, which are often accompanied by functional and cognitive impairment, and significant disability.^1^ Worldwide the illness affects over 1% of the general population.^1^ Despite substantial improvement in diagnosis and management it remains one of the most common causes of disability.^1^

During childhood and adolescence personality takes form and individuation occurs, while dysfunctional personality traits and interpersonal difficulties may emerge for some.^2^ Personality disorder (PD) occurs when an individual's personality structure inhibits them from achieving adaptive solutions to universal life tasks.^3^ Such tasks include difficulties forming integrated representations of oneself and the failure to develop intimate and cooperative personal and occupational relationships.^3^ Depending on the population sampled,^4–6^ the assessments administered^4–9^ and the symptomatic state at the time of assessment,^4–6,8,9^ the prevalence estimates for PD in people with BD range almost ten-fold from 9% to 89%.^4,10^

The relationship between PD and BD is nuanced and complex, with overlapping symptoms and ongoing debate regarding directionality.^11^ There are ten individual PDs listed in the Diagnostic and Statistical Manual of Mental Disorders (DSM) that are grouped into three clusters based on descriptive similarities.^12^ Self-harm, insecure attachment, identity issues and affective instability, such as the tendency to demonstrate variable and strong emotions, are common in people with certain PD types,^13^ but also overlap with some BD symptoms.^13^ Repeated BD affective episodes may contribute to the development of maladaptive behaviours, and the presence of chronic affective symptoms can appear as maladaptive personality traits, which may be attributed to BD itself.^6,14^ Comorbid antisocial PD is associated with an earlier onset of BD. People with BD and comorbid PD, compared to those with BD alone, show lower symptomatic recovery and functioning levels, increased hospitalisation, more severe mood disorder symptoms – including higher rates of suicidality and depression, and poorer long-term prognosis.^6,15–17^

A recent systematic review of pharmacological treatment outcomes of comorbid PD and mood disorders identified only one randomised controlled trial (RCT) in people with BD.^18^ This RCT found that antisocial PD did not significantly predict outcomes with acamprosate treatment in people with BD.^19^ In another retrospective study, people with comorbid PD demonstrated less compliance to treatment and poorer symptomatic recovery after the hospitalisation of mania.^14^ However, in a case-controlled study, Swartz et al.^20^ reported that people with BD and comorbid borderline PD had significantly greater improvement in their clinical impression and symptom severity scores after a combination of psychotherapies and pharmacotherapies, compared to those without borderline PD. Whether this characterises the effect of psychotherapy or the disorder itself remains uncertain. However, those with BD and comorbid borderline PD took significantly longer to achieve stabilisation than the BD-only group.^20^ Preston et al.^21^ trialled lamotrigine monotherapy for BD in a study in which borderline PD was retrospectively diagnosed. Following treatment, people with BD and comorbid borderline PD showed a 45% decrease in borderline personality symptom burden (compared with a 38% decrease for those with BD only). However, only 29% of those with comorbid borderline PD and BD were monotherapy responders, compared with 48% in the BD-only group.^21^

Overall, the available evidence underlining pharmacological treatment outcomes is limited, and there is a lack of rigorously designed treatment trials investigating the influence of PD on the treatment outcomes in people with BD. This is partly because people with PD may have been excluded in RCT designs or PD measurements were omitted.^18^ Additionally, researchers may find it difficult to engage with people with PD in clinical trials, or participants may not disclose personality psychopathology – potentially due to disruption in self-awareness or identity, or not perceiving traits as being maladaptive.^22^ With a substantial gap in pharmacological treatment outcomes and conflicting research evidence, there is a need to further investigate the impact of comorbid PD on treatment response to pharmacotherapies.

This study explored whether the presence of potentially maladaptive personality traits influenced treatment outcomes in people with BD, via a secondary data analysis of an RCT exploring adjunctive treatments for bipolar depression.^23^ The primary aim of this study was to determine whether participants with BD alone versus those with BD and above threshold personality symptoms (on a standardised questionnaire) differed in treatment outcomes during the course of the RCT. It was hypothesised that participants with above-threshold personality symptoms would have poorer treatment outcomes than those with BD alone.

## Material and Methods

### Overarching Trial Design

Data for this secondary analysis were derived from a 16-week, double-blind RCT investigating the efficacy of adjunctive N-acetylcysteine (NAC) or combination treatment (CT) for bipolar depression.^23^ This overarching trial study was approved by the relevant ethics committees at each site (Barwon Health, The Melbourne Clinic, Royal North Shore Hospital), was registered with ANZCTR (ACTRN12612000830897), and was conducted in accordance with good clinical practice guidelines. Participants provided written informed consent before being recruited into the trial. Participants were randomised to one of the three-arm treatment groups (NAC, NAC with nutraceuticals [CT)] or placebo). Full details of the treatment regimen and clinical outcomes are available at Berk et al.^23^

### Participants

A total of 181 participants were enrolled in the overarching trial. Participants were recruited across three trial sites in Sydney, Melbourne and Geelong, and attended clinical interviews every 4 weeks, with a post-discontinuation visit conducted at week 20. Participants were included if they met the DSM-IV criteria for BDI, BDII or BD not otherwise specified via the Mini-International Neuropsychiatric Interview Plus (MINI-Plus)^24^ and had moderate to severe depressive symptoms (a score of ≥20 on the Montgomery Åsberg Depression Rating Scale, MADRS).^25^ Participants were included in the current nested study if they also completed the Standardised Assessment of Personality – Abbreviated Scale **(**SAPAS) tool, administered at week 4 of the trial (*n *= 148).^26^ The SAPAS was administered at week 4 to reduce burden on participants as the baseline assessment was lengthy.

### Outcome Measures

The SAPAS is a validated, 8-item screening measure to assess PD presence in clinical populations (total score range 0–8).^26^ Participants were categorised as having either BD alone or BD with comorbid PD, using a SAPAS score of ≥3 to indicate maladaptive PD traits. This method has been used in similar studies investigating the influence of PD on mood disorder outcomes.^27^

The MADRS total score was the primary interviewer-rated outcome for the overarching trial.^25^ Bipolar depression symptom severity was also measured using interviewer-rated Bipolar Depression Rating Scale (BDRS).^28^ Manic and hypomanic symptoms were measured with the Young Mania Rating Scale (YMRS).^29^ The Clinical Global Impression – Improvement Scale (CGI-I)^30^ and The Clinical Global Impression – Severity (CGI-S)^31^ were used to measure symptom severity and improvement, respectively. Perceived patient improvement was measured via the Patient Global Impression-Improvement (PGI-I) scale.^30^

Functioning was measured using the Social and Occupational Functioning Assessment Scale (SOFAS),^32^ and the Longitudinal Interval Follow-up Evaluation – Range of Impaired Functioning Tool (LIFE-RIFT).^33^ The Quality of Life Enjoyment and Satisfaction Questionnaire-Short Form (Q-LES-Q-SF) was used to assess perceived quality of life across a range of life areas.^34^

### Statistical Analyses

Statistical analysis was conducted using IBM^®^ SPSS^®^ Statistics for Windows, Version 28.^35^ The data were analysed using a modified intention-to-treat protocol and included all randomised participants who had a SAPAS score and post-baseline data. The differences in baseline characteristics between groups (i.e., BD alone and BD with PD) were compared via chi-square for non-continuous data and independent samples *t*-test for continuous data analyses.

The Generalised Estimated Equation (GEE) model was used to assess whether the presence of above-threshold personality symptoms (*PD status*) predicted differences in treatment outcome measures (MADRS, BDRS, YMRS, CGI-I, CGI-S, PGI-I, SOFAS, LIFE-RIFT and Q-LES-Q-SF) when compared with participants with BD alone. The GEE models assumed normal distribution for the outcome, an identity link function and used unstructured robust covariance pattern to account for within-participants autocorrelation. This methodology was used previously on the current RCT in a study exploring physical activity as a predictor of treatment outcomes.^36^ Each outcome measure was explored individually and between each of the treatment groups (NAC, CT or placebo) at baseline, week 16 and week 20 follow up.

The GEE models included the main effect of *treatment group*, *time*, *PD status*, all two-way interactions and the three-way interaction of *PD status*, *treatment group* and *time*. Estimation of the three-way interactions of *PD status*, *treatment group* and *time* were initially explored, however, due to the small sample size and low statistical power within each treatment group, the three-way way interactions were not robust and thus were not meaningful. Therefore, the two-way interactions of *PD status* by *time* (i.e., follow-up trajectories of outcome measures in NAC, CT or placebo) or *PD status* by *treatment group* at week 16 (end of trial treatment) or week 20 (post-discontinuation) relative to baseline were included in the reported models (i.e., effect modification at week 16, and week 20). These models allowed for the exploration of the relationships between individual treatment outcome measures and the presence or absence of comorbid PD over time and between treatment groups, and of PD as a non-specified effect modifier. Investigating PD status as a non-specified predictor explored whether PD status has an influence on the overall sample, independent of assessment time or treatment group. The Wald χ^2^ statistic for each model parameter, and associated 95% confidence intervals (CIs), β Coefficients and *p* values were reported. For significant outcomes identified in the two-way interactions (*PD status* by *time* or *PD status* by *treatment group*) post hoc analysis from the GEE was conducted to determine which means were significantly different.

## Results

### Participant Characteristics

**Figure 1.**
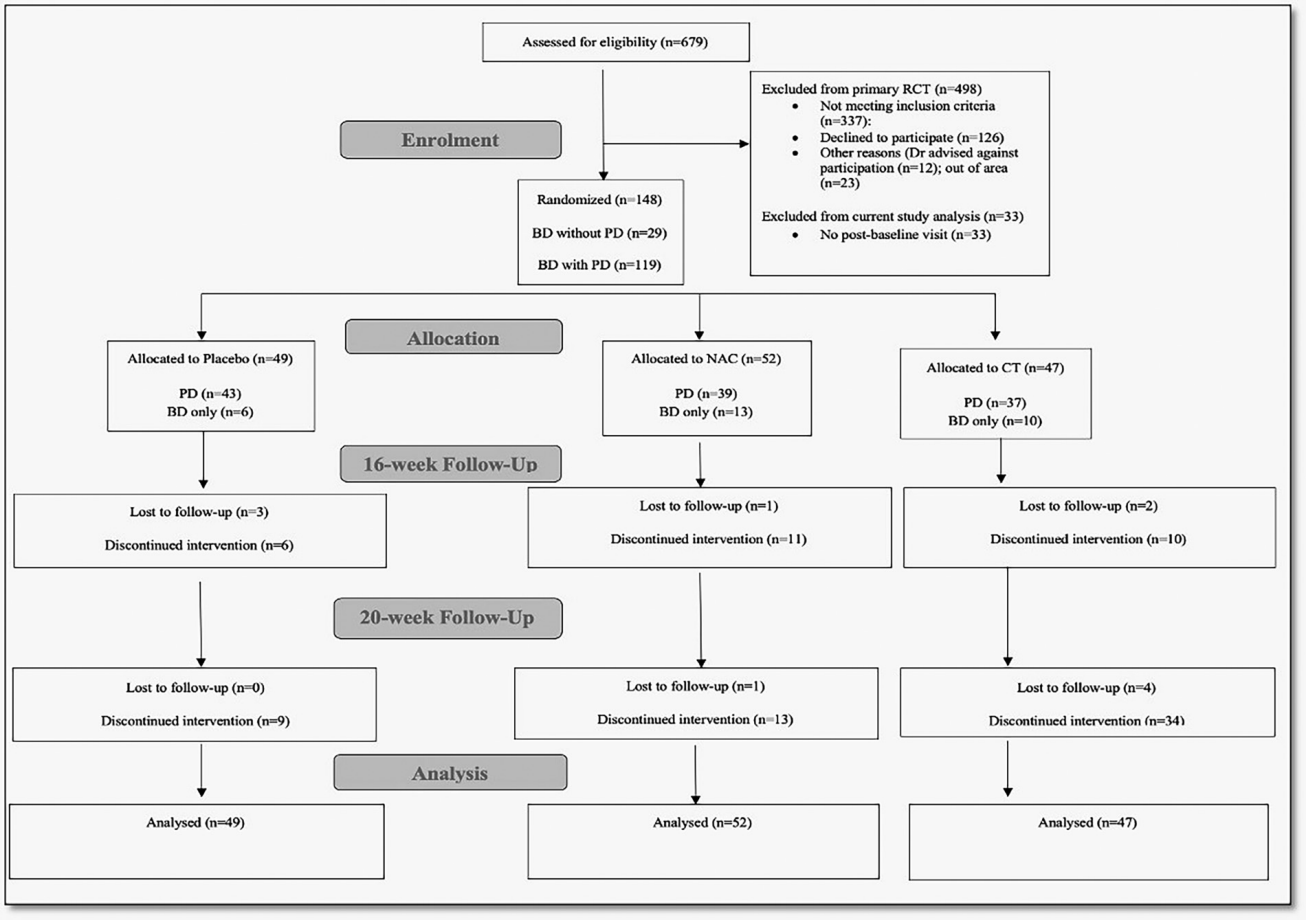
Participant flow diagram.

Of the original 181 participants in the trial, 33 were excluded from this study due to no available post-baseline data (including SAPAS; Figure 1). Therefore 148 participants were included for analysis, of which 80% (*n *= 119) had a SAPAS score ≥3 and were classified as the PD group. Participant characteristics can be seen in Table 1. The median age of participants was mid to late 40s and 35% of the cohort were male.

**Table 1.** Baseline Demographic Characteristics of Participants with Bipolar Disorder Alone and Participants with Comorbid Personality Disorder.

Demographic/diagnostic groups	BD alone (*n* = 29)	BD with PD (*n* = 119)	Effect size (95% CI) Cohen's *d*/Cramer's *V*	*p*
Mean age (SD)	48.83 (12.73)	45.47 (12.18)	0.27 (−0.13–0.68)	0.19
Males, *n* (%)	9 (31)	43 (36)	0.04	0.61
Treatment groups (n) Placebo, NAC, CT	6,13,10	43,39,37	0.14	0.26
Duration of Illness (years), mean (SD)	24.97 (11.92)	25.57 (11.94)	−0.05 (−0.45–0.36)	0.81
Number of hospitalisations, *n* (%)			0.13	0.46
None	9 (31.0)	37 (31.1)		
One	4 (13.8)	25 (21.0)		
2–10	12 (41.4)	50 (42.0)		
>10	4 (13.8)	7 (5.9)		
Number of depressive episodes, *n* (%)			0.14	0.29
1–10	7 (25.9)	17 (15.7)		
10–20	6 (22.2)	18 (16.7)		
20+	14 (51.9)	73 (67.6)		
Suicide attempts, *n* (%)			0.11	0.61
0	16 (55.2)	57 (47.9)		
1	6 (20.7)	25 (21.0)		
2–4	4 (13.8)	29 (24.4)		
5+	3 (10.3)	8 (6.7)		
Psychiatric comorbidity (MINI-plus) – yes (%)				
Bipolar disorder without psychotic features	8 (27.6)	33 (27.7)	0.02	0.99
Bipolar I current	13 (44.8)	51 (42.9)	0.02	0.85
Bipolar I past	16 (55.2)	55 (46.2)	0.07	0.39
Bipolar II current	9 (31.0)	39 (32.8)	0.01	0.86
Bipolar II past	8 (27.6)	46 (38.7)	0.09	0.27
Phobias and panic disorder	8 (27.6)	61 (51.3)	0.19	0.02*
Generalised anxiety disorder current	12 (41.4)	52 (43.7)	0.02	0.82
Obsessive-compulsive disorder current	1 (3.4)	22 (18.5)	0.16	0.05*^
Post-traumatic stress disorder current	5 (17.2)	18 (15.1)	0.02	0.78^
Lifetime alcohol dependence and/or abuse	11 (37.9)	65 (55.1)	0.14	0.10
Lifetime substance dependence and/or abuse	6 (20.7)	47 (39.5)	0.16	0.06
Suicidality risk [*n*(%)] – current			0.09	0.72
No risk	8 (27.6)	23 (19.3)		
Low	10 (34.5)	41 (34.5)		
Moderate	4 (13.8)	16 (13.5)		
High	7 (24.1)	39 (32.8)		
Baseline medications [yes (%)]				
Antidepressants	17 (58.6)	67 (56.3)	0.02	0.82
Benzodiazepines	7 (24.1)	22 (18.5)	0.06	0.49
Antipsychotic	16 (55.2)	71 (59.7)	0.04	0.66
Mood stabiliser	21 (72.4)	81 (68.1)	0.04	0.65
Baseline symptoms status and functioning, mean (SD)				
MADRS	27.59 (6.00)	29.44 (5.37)	−0.33 (−0.74–0.07)	0.11
BDRS	22.76 (6.63)	25.46 (6.37)	−0.42 (−0.83–−0.01)	0.05*
YMRS	2.93 (4.04)	3.61 (3.19)	−0.20 (−0.61–0.21)	0.33
CGI-S	4.45 (0.95)	4.58 (0.80)	−0.16 (−0.57–0.25)	0.44
SOFAS	57.69 (10.13)	56.37 (9.95)	0.13 (−0.27–0.54)	0.52
LIFE-RIFT	12.79 (2.47)	14.52 (2.72)	−0.65 (−1.06–−0.23)	0.002*
Q-LES-Q-SF	44.77 (15.78)	40.25 (12.10)	0.35 (−0.06–0.76)	0.09

BD: bipolar disorder; BDRS: Bipolar Depression Rating Scale; CGI-S: Clinical Global Impression – Severity scale; LIFE-RIFT: Range of Impaired Functioning Tool; MADRS: Montgomery Åsberg Depression Rating Scale; MINI-Plus: MINI-international neuropsychiatric interview plus; PD: personality disorder; PGI – I: Patient Global Impression-Improvement subscale; Q-LES-Q-SF: Quality of Life and Enjoyment Satisfaction Questionnaire; SD: standard deviation; SOFAS: Social And Occupational Functioning Assessment scale; YMRS: Young Mania Rating Scale; NAC: N-acetylcysteine; CT: combination treatment.

*Significant value when p < 0.05. ^ Chi-square unable to be computed due to few data (not meeting the assumption of a minimum of 5 cells, therefore the Fishers exact value is used).

 

**Table 2.** Mean Scores (SD) at Baseline, Week 16 and Week 20 on Rating Scales in Participants with Bipolar Disorder Along and Participants with Bipolar Disorder and Comorbid Personality Disorder.

	Bipolar disorder alone									Bipolar disorder with PD								
	Baseline			Week 16			Week 20			Baseline			Week 16			Week 20		
Scale/ Treatment Groups	Placebo n = 6	NAC n = 13	CT n = 10	Placebo n = 5	NAC n = 10	CT n = 6	Placebo n = 5	NAC n = 10	CT n = 7	Placebo n = 43	NAC n = 39	CT n = 37	Placebo n = 35	NAC n = 30	CT n = 29	Placebo n = 35	NAC n = 27	CT n = 25
MADRS	23.83 (3.31)	29.23 (6.19)	27.70 (6.43)	11.40 (7.67)	15.80 (11.87)	11.50 (7.82)	18.60 (13.12)	16.2 (8.23)	16.29 (8.86)	29.30 (5.51)	29.33 (4.96)	29.70 (5.75)	16.46 (10.79)	14.77 (9.55)	17.10 (10.21)	17.63 (10.96	16.07 (10.57)	12.28 (8.43)
BDRS	21. 83 (7.33)	24.39 (7.25)	21.20 (5.45)	7.80 (7.53)	15.00 (11.97)	8.67 (3.20)	16.20 (12.21)	16.11 (8.61)^a^	16.00 (10.26)	24.28 (7.44)	26.31 (4.84)	25.87 (6.42)	14.58 (10.15)^c^	12.93 (9.21)^f^	14.89 (9.26)^g^	14.78 (9.29)^d^	14.11 (8.65)	10.78 (7.64)^h^
YMRS	2.00 (1.67)	2.15 (2.97)	4.50 (5.78)	2.60 (2.41)	2.90 (5.20)	6.33 (6.89)	2.80 (2.17)	3.00 (4.87)^a^	7.29 (7.70)	3.41 (3.77)	3.68 (2.79)	3.78 (2.92)	2.96 (4.10)^c^	3.09 (3.11)^f^	3.48 (3.69)^g^	3.82 (4.00)^c^	3.65 (3.89)	2.17 (2.11)^h^
CGI-I				1.60 (0.55)	2.30 (1.25)	1.83 (0.75)	2.00 (1.22)	2.30 (0.95)	2.57 (1.27)				2.51 (1.15)	2.29 (0.94)	2.39 (0.99)^g^	2.71 (1.30)	2.44 (0.97)	1.96 (0.73)
CGI-S	4.00 (0.63)	4.62 (0.87)	4.50 (1.18)	2.20 (1.30)	3.20 (1.75)	2.83 (1.17)	2.80 (1.64)	2.90 (1.10)	3.14 (1.35)	4.49 (0.70)	4.64 (0.67)	4.62 (1.00)	3.14 (1.35)	3.07 (0.91)	3.21 (1.20)^g^	3.20 (1.32)	3.37 (1.33)	2.60 (1.26)
PGI-I				2.00 (0.71)	2.30 (1.25)	2.17 (1.47)	1.80 (0.84)	2.00 (0.87)^a^	2.00 (0.82)				2.43 (1.17)	2.53 (1.07)	2.36 (1.31)^g^	2.77 (1.28)^e^	2.70 (1.27)	2.20 (1.08)
LIFE-RIFT	12.83 (2.93)	13.23 (2.20)	12.20 (2.66)	9.80 (3.03)	10.30 (4.60)	8.83 (2.48)	12.00 (3.67)	10.33 (4.00)^a^	9.86 (4.10)	13.91 (2.85)	14.59 (2.72)	15.16 (2.49)	10.97 (3.95)^c^	11.00 (3.12)^f^	10.72 (3.74)^g^	11.13 (3.95)^d^	11.33 (3.63)	10.17 (4.20)^h^
SOFAS	59.83 (8.23)	56.70 (10.97)	57.70 (10.81)	75.80 (7.82)	66.80 (10.19)	72.17 (12.01)	70.00 (11.75)	69.88 (16.13)^b^	69.71 (13.78)	57.09 (8.50)	56.18 (9.46)	55.73 (12.02)	69.46 (10.95)^c^	67.66 (11.75)^f^	68.92 (15.27)^g^	68.21 (11.12)^c^	67.74 (12.98)	72.72 (17.79)
Q-LES- Q-SF	41.37 (20.12)	43.13 (17.46)	48.93 (10.59)	66.07 (9.36)	57.14 (18.46)	67.56 (15.50)	48.21 (26.31)	55.71 (17.00)	63.01 (11.79)	42.94 (11.18)	40.11 (10.67)	37.26 (13.99)	58.09 (17.10)^e^	57.43 (16.07)	56.22 (16.83)	56.75 (19.06)^d^	56.55 (17.59)	61.93 (17.64)

BDRS: Bipolar Depression Rating Scale; CGI-I: Clinical Global Impression – Improvement scale; CGI-SI: Clinical Global Impression – Severity scale; LIFE-RIFT: Range of Impaired Functioning Tool; MADRS: Montgomery Åsberg Depression Rating Scale; PGI – I: Patient Global Impression-Improvement subscale; Q-LES-Q-SF: Quality of Life and Enjoyment Satisfaction Questionnaire; SOFAS: Social And Occupational Functioning Assessment Scale; YMRS: Young Mania Rating Scale; NAC: N-acetylcysteine; CT: combination treatment.

^a^ *n* = 9.

^b^ *n* = 8.

^c^ *n* = 33.

^d^ *n* = 32.

^e^ *n* = 34.

^f^ *n* = 29.

^g^ *n* = 28.

^h^ *n* = 23.

### Impact of PD Status by Treatment Group and by Time

The mean scores for each outcome variable for each time point and treatment group are listed in Table 2. The PD status by treatment group interactions were not significant for any outcome measures from baseline to week 16 or week 20 (see Table 3). A significant interaction was found between PD status by time for the PGI-I (χ2(1) = 11.46, *p *= 0.02) at 20 weeks. Post-hoc analyses from the GEE showed a significant difference at week 20 between comorbid PD [2.58 (±1.23)] and BD alone [1.95 (±0.80)], *B* = 0.93, *df *= 1, χ2(1) = 10.01, *p *= 0.006]. There were no further significant two-way interactions of PD status by time from baseline to week 16 or week 20 (see Table 3).

**Table 3.** Treatment Outcomes from Baseline, Week 16 and Week 20 and Two-way Interactions of Personality Disorder by Time or Treatment Group from Participants with Bipolar Disorder and Participants with Bipolar Disorder and Comorbid Personality Disorder.

Disorder groups							Interactions in Week 16 (End of treatment)				Interactions in Week 20 (Follow-Up)			
	Bipolar disorder alone			Bipolar disorder with comorbid personality disorder			Two-way interactions of personality disorder by time		Two-way interactions of personality disorder by treatment group		Two-way interactions of personality disorder by time		Two-way interactions of personality disorder by treatment group	
Trial weeks/ outcome measures	Baseline	Week 16	Week 20	Baseline	Week 16	Week 20	β Interaction (95% CI)	χ^2^ (1), *P* value	β Interaction (95% CI)	χ^2^ (1), *P* value	β Interaction (95% CI)	χ^2^ (1), *P* value	β Interaction (95% CI)	χ^2^ (1), *P* value
MADRS Total Means (*SD*)	26.92 *(5.31)*	12.9 (*9.12*)	17.03 (*10.07*)	29.45 (*5.41*)	16.11 (*10.18*)	15.33 (*9.99*)	1.99 (−0.31 _to_ 4.29)	0.93, *0.92*	2.50 (−0.91 _to_ 5.83)	0.50, *0.78*	2.00 (−0.31 _to_ 4.29)	3.55, *0.62*	1.94 (−2.08 _to_ 5.96)	0.44, *0.80*
BDRS Total Means (*SD*))	22.47 (*12.39*)	10.49 (*7.57*)	16.10 (*10.36*)	25.48 (*6.23*)	14.13 (*9.54*)	13.22 (*8.53*)	2.99 (−0.41 _to_ 5.56)	1.13, *0.89*	3.20 (−0.09 _to_ 6.49)	0.58, *0.75*	2.99 (0.41 _to_ 5.56)	8.50, *0.13*	2.51 (−1.56 _to_ 6.57)	0.39, *0.83*
YMRS Total Means (*SD*)	2.88 (*3.47*)	3.94 (*4.83*)	4.36 (*4.91*)	3.62 (*3.16*)	3.17 (*3.63*)	3.21 (*3.26*)	0.73 (−0.78 _to_ 2.24)	2.67, *0.61*	0.28 (−1.19 _to_ 1.75)	0.07, *0.97*	0.73 (−0.78 _to_ 2.24)	4.35, *0.50*	0.39 (−1.02 _to_ 1.81)	0.52, *0.77*
^CGI-I Total Means (*SD*)		1.91 (*0.85*)	2.29 (*1.15*)		2.40 (*1.03*)	2.37 (*1.00*)	0.21 (−0.70 _to_ 0.29)	3.52, *0.32*	0.17 (−0.23 _to_ 0.57)	0.74, *0.69*	−0.21 (−0.70 _to_ 0.29)	3.93, *0.42*	0.27 (−0.17 _to_ 0.71)	2.02, *0.37*
CGI-S Total Means (*SD*)	4.37 (*0.89*)	2.11 (*1.41*)	2.95 (*1.36*)	4.58 (*0.79)*	3.14 (*1.15*)	3.06 (*1.31*)	0.17 (−0.20 _to_ 0.53)	1.78, *0.78*	0.44 (−0.06 _to_ 0.93)	1.68, *0.43*	0.17 (−0.20_to_ 0.53)	1.82, *0.87*	0.43 (−0.16 _to_ 1.02)	1.56, *0.46*
^PGI-I Total Means (*SD*)		2.16 (*1.14*)	1.93 (*0.84*)		2.44 (*1.19*)	2.56 (*1.21*)	−0.33 (−0.78 _to_ 0.12)	3.42, *0.33*	0.24 (−0.10 _to_ 0.58)	1.75, *0.42*	−0.33 (−0.78 _to_ 0.12)	11.46, 0.02*	0.38 (0.01 _to_ 0.75)	2.25, *0.33*
SOFAS Total Means (*SD*)	58.08 (*10.00*)	71.59 (*13.01*)	69.86 (*13.89*)	56.33 (*10.00*)	68.68 (*12.65*)	69.56 (*12.96*)	−1.53 (−5.57 _to_ 2.52)	2.01, *0.73*	−3.00 (−7.74 _to_ 1.82)	0.11, *0.95*	−1.53 (−5.57 _to_ 2.52)	3.05, *0.69*	−2.78 (−8.16 _to_ 2.60)	0.17, *0.92*
LIFE-RIFT Total Means (*SD*)	12.75 (*2.60*)	9.64 (*3.71*)	10.73 (*3.92*)	14.55 (*2.69*)	10.84 (*3.60*)	10.88 (*3.92*)	1.85 (0.81 _to_ 2.89)	3.92, *0.42*	0.82 (−0.85 _to_ 2.49)	0.38, *0.83*	1.85 (0.81 _to_ 2.88)	5.69, *0.34*	0.57 (−1.27 _to_ 2.40)	0.50, *0.78*
Q-LES-Q-SF Total Means (*SD*)	44.48 (*16.06*)	63.59 (*14.44*)	55.65 (*18.36*)	40.10 (*11.95*)	57.25 (*16.66*)	58.41 (*18.09*)	−4.94 (−11.15 _to_ 1.27)	0.50, *0.97*	−2.33 (−10.93 _to_ 6.27)	0.82, *0.67*	−4.94 (−11.15 _to_ 1.27)	6.80, *0.24*	−0.70 (−10.59 _to_ 9.20)	0.86, *0.65*

BDRS: Bipolar Depression Rating Scale; CGI-I: Clinical Global Impression – Improvement scale; CGI-SI: Clinical Global Impression – Severity scale; LIFE-RIFT: Range of Impaired Functioning Tool; MADRS: Montgomery Åsberg Depression Rating Scale; PGI – I: Patient Global Impression-Improvement subscale; Q-LES-Q-SF: Quality of Life and Enjoyment Satisfaction Questionnaire; SOFAS: Social And Occupational Functioning Assessment Scale; YMRS: Young Mania Rating Scale; NAC: N-acetylcysteine; CT: combination treatment. *Significant value when *p* < 0.05, remain significant after Bonferroni correction for multiple comparisons across the follow-ups time points (i.e., *p*-value <.025). ^CGI-I and PGI-I were captured at Week 4 as they have no baseline data due to these outcomes being improvement scores over time. Two-way interactions of PD by time (i.e., follow-up trajectories of outcome measures in NAC, CT or placebo) or PD by treatment group at week 16 (end of trial treatment) or week 20 (post-discontinuation) relative to baseline were included in the models (i.e., effect modification at week 16, and week 20). These models allowed for the exploration of the relationships between individual treatment outcome measures and the presence or absence of comorbid PD over time and between treatment groups or as PD as an effect modifier.

### PD Status as a Non-specified Predictor

For BDRS (χ^2^(1) = 4.38, *p *= 0.04), LIFE-RIFT (χ^2^(1) = 7.69, *p *= 0.01), and Q-LES-Q-SF (χ^2^(1) = 3.89, *p *= 0.05), the presence of PD was associated with poorer scores for those outcome measures from baseline to week 16, regardless of timepoint or received treatment (see Table 4). No significant effect of PD status was found on any outcome measure for the post-discontinuation visit.

**Table 4.** Comorbid Personality Disorder on the Influence on Bipolar Disorder as a Non-specified Predictors of Treatment Outcomes from Baseline to Week 16 and Week 20.

Baseline to Week 16			Baseline to Week 20	
Predictor	Model-based mean difference (95% CI)	χ^2^ (1), *P*-value	Model-based mean difference (95% CI)	χ^2^ (1), *P*-value
MADRS	1.61 (−0.67 to 3.89)	1.91, *0.17*	1.24 (−0.98 to 3.49)	1.19, *0.28*
BDRS	2.44 (−0.15 to 4.72)	4.38, 0.04*	1.65 (−0.65 to 3.96)	1.98, *0.16*
YMRS	0.25 (−0.94 to 1.44)	0.17, *0.69*	0.11 (−1.14 to 1.35)	0.03, *0.87*
^CGI-I	0.06 (−0.23 to 0.36)	0.18, *0.67*	0.04 (−0.26 to 0.34)	0.07, *0.79*
CGI-S	0.13 (−0.17 to 0.43)	0.72, *0.40*	0.11 (−0.19 to 0.40)	0.51, *0.48*
^PGI-I	−0.30 (−0.34 to 2.78)	0.04, *0.85*	0.05 (−0.24 to 0.35)	0.12, *0.73*
SOFAS	−0.69 (−5.39 to 4.01)	0.08, *0.77*	3.50 (−4.24 to 11.24)	0.79, *0.38*
LIFE-RIFT	1.35 (0.40 to 2.30)	7.69, 0.01*	1.37 (−7.05 to 9.78)	0.10, *0.75*
Q-LES-Q-SF	−4.59 (−9.14 to −0.03)	3.89, 0.05*	−3.69 (−8.41 to 1.03)	2.35, *0.13*

BDRS: Bipolar Depression Rating Scale; MADRS: Montgomery Åsberg Depression Rating Scale; CGI-I: Clinical Global Impressions and Improvement Scale; CGI-S: Clinical Global Impressions and Severity Scales; PGI-I: Patient Global Impressions–Improvement scale; SOFAS: Social and Occupational Functioning Assessment Scale; LIFE-RIFT: Range of Impaired Functioning Tool; Q-LES-Q-SF: Quality of Life Enjoyment and Satisfaction Questionnaire-Short Form; YMRS: Young Mania Rating Scale. *Significant value when *p* < 0.05. ^CGI-I and PGI-I were captured at Week 4 as they have no baseline data due to these outcomes being improvement scores over time.

## Discussion

This novel study involved secondary analysis of data from an RCT to determine whether treatment outcomes differed in participants with BD with and without potentially maladaptive personality traits.^23^ Surprisingly, on most measures, presence of PD traits did not affect differences between treatments or in changes in clinical ratings over time. The only significant outcome was a patient-rated outcome measure, the PGI. Given this study was exploratory and the overarching study was not powered for personality outcomes, these finding must be interpreted with caution. However, the study highlights the importance of patient-rated metrics like the PGI, alongside clinical measures in clinical trials and their potential value in the assessment of people with PD. The presence of PD traits based on the SAPAS threshold was also a significant non-specific predictor of poorer outcomes on multiple outcomes including the BDRS (bipolar depression), LIFE-RIFT (psychopathology/functioning) and Q-LES-Q-SF (quality of life), regardless of time or treatment group.

### PD Did Not Impact on Treatment Outcomes in an RCT

It is pertinent to note that the interpretation of these findings is in the context of an RCT, where participant response may not reflect the true outcomes of real-world treatment setting by virtue of its clinical setting. Furthermore, the participants were experiencing moderate to severe depression symptoms when they commenced the trial. This may have contributed to the high prevalence (80%) of PD traits based on the SAPAS threshold from, for example, a bias towards negative self-perception. This may be further exacerbated by other comorbid mental health conditions in the sample, such as anxiety disorders (e.g., PTSD was present in 15% of the sample).^37^

However, the lack of significant interactions between PD and treatment outcomes in BD is consistent with a recent systematic review by Kavanagh et al.^18^ on the influence of comorbid PD on mood disorders. It was found that people with mood disorders and comorbid PD did not have significant differences in treatment remission and/or outcome, compared to those with mood disorders alone.^18^ However, only one RCT was included in the review, assessing acamprosate treatment in people with BD, which found antisocial PD did not significantly impact outcomes.^19^ Similarly, Kavanagh et al.^22^ found that in those with mood disorder symptoms, personality pathology did not substantively influence pharmacology treatment outcomes.^22^ Overall, there is a lack of studies exploring the efficacy of anticonvulsants and mood stabilisers on BD by PD status.^18^ Similarly, the potential role of adjunctive nutraceuticals, such as the therapy used in this study, for treatment of PD is unclear. A systematic review has identified a potential role for the use of marine omega-3 fatty acids in people with borderline PD,^38^ however, less is known about the role of nutraceuticals in comorbid BD and PD. Overlapping symptoms and pathophysiology of BD and PD^39^ may mean that any effective adjunctive nutraceuticals identified for BD may also have a role to play in PD treatment.

There was a high rate of participants with above-threshold personality symptoms. Future research should investigate the effects of PD in pharmacological RCT's for an extended period of time to clarify the effects of treatment outcomes, since few studies have examined outcomes beyond 12 weeks. Despite the lack of evidence of treatment response differences in pharmacological interventions,^22^ additional investigations exploring both the two-way and three-way interactions of PD over time and treatment are warranted. This will help further understand the potential mediating role of PD in pharmacological treatment and its outcomes.

### Patient Perception of Improvement

Even though the primary hypothesis was not supported, the study does provide some novel findings. The presence of maladaptive traits appeared to influence patient perception of clinical improvement (shown on PGI-I scores). Cluster B and cluster C PDs are commonly seen in people with BD^40^; the DSM-5 denotes those with cluster B to be more emotional, dramatic or erratic and those with cluster C to be more anxious or fearful.^12^ In addition, those with avoidant PD display patterns of hypersensitivity to negative evaluation or a predisposition to feelings of inadequacy.^12^ Furthermore, people with borderline-type personalities in particular often have alexithymia, making it difficult to read their own emotions. Borderline PD has been noted to be both clinically and neurobiologically distinct from BD.^41,42^ Clinically, the management of PDs is often complicated by non-concordance between clinician and patient perceptions.^43^ These factors may hinder accurate self-reporting and may have influenced the significant PGI-I results found in this study.

This study presented discordance between results on the PGI-I and CGI-I, the former being significantly different between those with BD alone and those with comorbid PD at week 20. This contrasts with the findings of Mohebbi and colleagues that the CGI-I and PGI-I measures generally have high agreement with each other,^44^ however PD was not measured in their study. Patient experiences are valid and informative in research and clinical outcome assessment.^44^ In the context of this study, people with PD perceived less improvement overall compared to those with BD alone, even if clinician ratings indicated global improvement from baseline.

PD status was a non-specified predictor for BDRS, LIFE-RIFT and Q-LES-Q-SF outcomes from baseline to week 16. A longitudinal study by Ng et al.^45^ investigated the impact of PD symptom severity on the progression of bipolar spectrum disorders – more severe PD symptoms were associated with an increased risk of both major depressive and hypomanic episodes.^45^ PD traits such as aggression and impulsivity that are associated with more stressful life events (i.e., trauma, abuse) are consequential potential precursors to the development of mood episodes.^46–49^ These factors may progressively exacerbate PD symptoms and predict the course of bipolar spectrum disorders.^45,47,49,50^

With respect to functioning, Loftus and Jaeger^51^ showed the presence of PD or maladaptive trait scores resulted in more impaired functioning in areas of residential, occupational, leisure and social outcomes. In addition to this, poorer functioning was associated with higher levels of residual manic or depressive symptoms that were attributed to a greater number of maladaptive traits present.^51^

With regard to quality of life, Kavanagh et al.^52^ investigated the influence of PD on quality of life in women with other psychiatric disorders.^52^ Women with comorbid PD and other psychiatric disorders perceived themselves to have poorer psychological health than those with other psychiatric disorders alone or compared to control participants.^52^ People with PD may be more likely to make poorer health choices such as drinking or smoking,^53^ live solitary lifestyles or engage in turbulent relationships which lead to a lack of social connectedness.^52^

### Strengths and Limitations

This study was seated in the context of a well-designed, double-blind RCT which provided robust clinical trial data. However, the overarching trial was not statistically powered for the present analyses, and the number of participants in the non-PD group was small, and as such differences in treatment outcomes could not be robustly ascertained for participants with and without potentially maladaptive personality traits. Furthermore, PD grouping was based on a screening questionnaire. Despite good predictive validity in clinical populations when compared with a gold standard diagnostic measure,^26^ the SAPAS-based categorisation resulted in most of the sample (80%) being categorised as having maladaptive personality traits. This not only reduced the capacity to detect between-group differences but also brings up the question of whether the nature of the overall sample (i.e., people with BD who are currently depressed) contribute to the elevated prevalence seen. Clinical trials also may carry the risk of selecting people who do poorly in primary and secondary care and this might influence case mix and hence outcomes. The fact that the trial did not have robust between-group differences in outcomes may decrease the likelihood of detecting moderating influences. The GEE model did not correct for multiple comparisons, therefore increasing the chance of a Type 1 error; the positive finding on the PGI needs to be regarded therefore as hypothesis generating. The measurement of PD was assessed via the SAPAS as opposed to the SCID-II, which does not allow for the identification of specific PD types. The SCID-II could have provided useful information for specific PD traits and outcomes, and whether this study has underrepresented or overrepresented specific clusters.^52^ PD was only measured at one-time point in the study (week 4) – therefore the study could not determine whether the endorsement of PD items in the SAPAS changed during the RCT.^52^ Although it can be argued that there would be an expected stability of PD symptoms, administering the SAPAS at a different timepoint (week 4) than the baseline assessment increases the chances of the scores being influenced by state effects. As the questionnaire was not readministered at a later time point, we also cannot determine whether there was a change in PD symptoms as there was an improvement in BD symptoms at the end of the trial.

### Conclusion and Future Directions

This exploratory study found that the presence of maladaptive personality traits did not affect change over time or between treatment groups, except for the post-discontinuation visit patient time point based on the PGI-I. Although the perceived improvement of those with PD may not necessarily align with a clinician's judgement, it is important to include patient-centric measures in future studies. The presence of PD was associated with poorer outcomes overall on several metrics including the measures of bipolar depression, functioning and quality of life. These results echo previous literature where those with comorbid PD have poorer symptom recovery, functioning and quality of life due to the presence of maladaptive traits disrupting treatment efficacy or exacerbating BD symptoms.^18,22,52^ In addition, omitting or failing to report on PD groups in study analyses can miss a key study influence by potentially omitting a key moderator and/or mediator of results.^18^ Future trials should routinely measure personality and consider stratifying for PD's in order to investigate how different PD subtypes may influence treatment outcomes. More robust studies are needed to evaluate the efficacy of pharmacological interventions in the presence of PD,^18^ to improve treatment outcomes.
